# Rhinoplastic and Cheiloplastic Operations

**Published:** 1841-05

**Authors:** J. Pancoast

**Affiliations:** Professor of Anatamy in Jefferson Medical College, one of the Surgeons of the Philadelphia Hospital, &c.


*Rhinoplastic and Cheiloplastic Operations.* By J. Pancoast, M. D., Professor of Anatamy in Jefferson Medical College, one of the Surgeons of the Philadelphia Hospital, &c.—Jno. Glover, æt. 50, a native of Bridgewater, in England, lost, seven years ago, as he states, by phagedenic ulceration, all the soft parts of the nose, the whole of the upper lip, the turbinated bones and septum of the nostrils. Though now in good health and robust, he appears an object of deformity so disgusting to himself, that he has voluntarily exiled himself from his family and home. On examination it was found that the teeth with their sockets had all disappeared from the upper jaw, and nearly all from the lower; a small strip of gum, a quarter of an inch wide, stretched across, and formed the only separation between the lower lip and the ends of the ossa nasi.

In consequence of the destruction of the upper lip and the sockets of the teeth, the chin came high up on the face, and the lower lip fell on the margin of the nasal cavern.

The cicatrization following the destruction of the upper lip and nose, had drawn in the angles of the mouth, so as to leave a round opening of not more than three quarters of an inch in diameter when the mouth was opened to the widest extent.

Jan. 20th, an operation was performed before the class of the Philadelphia Hospital, for the enlargement of the mouth and the reconstruction of the upper lip. The mouth was widened after the manner of Dieffenbach, for about five-eighths of an inch at each angle, by removing a slip of the muscle and integuments in front of the mucous membrane, and then dividing the membrane in two, and binding each half by suture over the raw edges of the new lip. A flap of integument was then raised from the muscles of each cheek, an inch in breadth and an inch and a half in length, and brought down and fastened together by hare lip suture in front of the gum, which had been previously made raw with the knife. The operation succeeded. The new lip became perfectly adherent to the gum, and presented a natural appearance.

March 27. Proceeded to the Rhinoplastic part of the operation. A flap of the integuments was raised from the forehead, nearly of a pyramidal shape, three inches long and three inches broad at the base, having an adherent strip raised from the scalp on the middle line an inch and a half long and five-eighths of an inch broad, to form the columna of the new nose. The flap was dissected up, remaining attached only by a pedicle between the eyebrows, and twisted so as to present its raw surface over the opening of the nose. The integuments of the forehead and scalp were then brought together with hare lip sutures, leaving only a small opening to fill up by granulation, the size of a twenty-five cent piece. A grove was cut along the sides of the nasal cavern into which the flap of the new nose was stitched. The new columna from the scalp was bent in, and fastened with hare lip pins to the upper margin of the gum, and the new lip formed on the 20th of January, and which were freshened with the knife for its insertion. The entire operation lasted a little over an hour, and was borne by the patient without a murmur. 31st. The dressings were removed, the wound in the forehead and scalp were found united, except in the middle space, by first intention. The new nose and the column had also united every where by first intention, and retained in a great degree the natural prominence. The patient suffered but little pain or inconvenience since the operation, and has the fairest prospect of having his hideous deformity entirely removed. The details of this case will be hereafter more fully given, as well as those of another case in which the operation was performed by Dr. Pancoast, on the 9th of January last. In the latter instance, the nose which had been lost by scrofulous ulceration, attended with a destruction of a considerable part of the hard palate, was rebuilt by flaps from the cheeks, and the operation has been so completely successful as to require a careful inspection to distinguish it from the natural organ.—*Med. Exam., April* 10.

